# FRET-based Visualization of PDGF Receptor Activation at Membrane Microdomains

**DOI:** 10.1038/s41598-017-01789-y

**Published:** 2017-05-09

**Authors:** Jihye Seong, Min Huang, Kyoung Mi Sim, Hyunbin Kim, Yingxiao Wang

**Affiliations:** 1 Neuroscience Program, University of Illinois, Urbana-Champaign, Urbana, IL 61801 USA; 2 Convergence Research Center for Diagnosis Treatment Care of Dementia, Korea Institute of Science and Technology (KIST), Seoul, 02792 South Korea; 3 Biological Chemistry Program, Korea University of Science and Technology (UST), Daejeon, 34113 South Korea; 4 Department of Converging Science and Technology, Kyung Hee University, Seoul, 02447 South Korea; 5 Department of Bioengineering, University of Illinois, Urbana-Champaign, Urbana, IL 61801 USA; 6 Department of Bioengineering, University of California, San Diego, CA 92093 USA

## Abstract

Platelet-derived growth factor receptor (PDGFR) senses extracellular growth factors and transfer the signals inside the cells regulating cell proliferation, migration and survival. It has been controversial at which membrane microdomains PDGFRs reside and how they control such diverse intracellular signaling pathways. Here, we developed a novel PDGFR biosensor based on fluorescence resonance energy transfer (FRET), which can detect the real-time PDGFR activity in live cells with high spatiotemporal resolutions. To study subcellular PDGFR activity at membrane microdomains, this PDGFR biosensor was further targeted in or outside lipid rafts via different lipid modification signals. The results suggest that, in response to PDGF stimulation, PDGFR activity is evenly distributed at different membrane microdomains, while integrin-mediated signaling events have inhibitory effects on the activation of PDGFR specifically located in lipid rafts but not outside rafts, implying the role of lipid microdomains as segregated signaling platforms.

## Introduction

Platelet-derived growth factor receptor (PDGFR) is activated by binding to its ligand PDGF, and involved in a variety of cellular processes, e.g. proliferation, migration, survival, and cancer development^1^. PDGF is secreted from many cell types including platelets, endothelial, epithelial, glial and inflammatory cells, and its receptor PDGFR is expressed in oligodendrocytes, fibroblasts, and vascular smooth muscle cells. Biochemically, PDGF is secreted as a homo- or heterodimer composed of A, B, C and D forms: AB, AA, BB, CC, and DD. These PDGF dimers bind to extracellular regions of PDGFR, triggering the dimerization of PDGFR. The ligand-bound PDGFR dimers induce the autophosphorylation of PDGFR, possibly through the conformational changes of the intracellular regions of these receptors. The phosphorylated PDGFRs then can initiate various downstream signaling events by recruiting SH2 domain-containing molecules such as Src kinase, Grb2, SHP2, PLCγ, Nck, and STAT^1, 2^.

To regulate such diverse signaling events, the activity of PDGFR is tightly regulated in space and time. Through its transmembrane domain, PDGFRs are located at the membrane which contains different microdomains such as lipid rafts. Lipid rafts are enriched in sphingolipids and cholesterol, and due to their physicochemical properties distinct from general membrane regions, it has been suggested that lipid rafts can function as segregated signaling platforms^3^. Traditionally, lipid rafts-related signaling has been studied by the separation of detergent-resistant membrane fractions, and by the treatment of methyl-β-cyclodextrin (MβCD), which disrupts the structure of lipid rafts. However, these biochemical methods are controversial, mainly because the separation of membrane fractions is found to be poor in specificity and dependent on the conditions and types of detergent^4^. Also, nonspecific effects of methyl-β-cyclodextrin have been reported in previous studies^5^. More importantly, it is difficult to monitor the highly dynamic features of lipid rafts with these traditional strategies^3, 6^.

Fluorescence resonance energy transfer (FRET)-based molecular biosensors have been developed to monitor local and dynamic activity of different signaling molecules, for example, Src, focal adhesion kinase (FAK), and Rho GTPases^7–10^. These biosensors are designed to alter their FRET levels as the target protein is activated. Furthermore, the biosensors can be targeted to the subcellular regions such as nucleus, plasma membrane, and endoplasmic reticulum, thus allowing the continuous visualization of the important signaling events at local regions *in situ* in live cells^6, 9^. Indeed, utilizing different lipid modification signals, i.e. acylation and prenylation, we have shown that the FRET biosensors can be successfully tethered in or outside lipid rafts, providing a powerful tool to study dynamic lipid rafts signaling events in live cells with high spatiotemporal resolutions^6, 8, 11–13^.

In fact, differential PDGF-related signaling events in and outside lipid rafts have been reported utilizing these subcellular targeted FRET biosensors^6^. For example, FRET-based Src biosensors reported a faster and stronger Src activation in lipid rafts upon PDGF stimulation^11^. In contrast, the PDGF-induced FAK activation was predominantly observed in lipid rafts^8^, and the Akt activation in response to PDGF was also faster and stronger in lipid rafts^12^. While PDGF-related signaling molecules are extensively studied, there has been no direct evidence on where PDGFRs reside in to regulate their functions. Here, we successfully developed a new FRET-based molecular biosensor detecting local PDGFR activities in and outside lipid rafts. Our results with the PDGFR FRET biosensors targeted at different microdomains showed that the significant PDGFR activation occurs both in and outside lipid rafts upon PDGF stimulation. Further results indicate that strong integrin-mediated signaling events have inhibitory effects on the PDGFR activity at lipid rafts, but not at non-raft regions. Therefore, our results with these new PDGFR FRET biosensors suggest that there are differential regulation processes of PDGFR activity at different membrane microdomains, which is dependent on integrin-mediated signals. These results suggest that lipid rafts can function as signaling platforms for the precise control of cellular functions.

## Results

### Development of FRET-based PDGFR Biosensor

**Figure 1:**
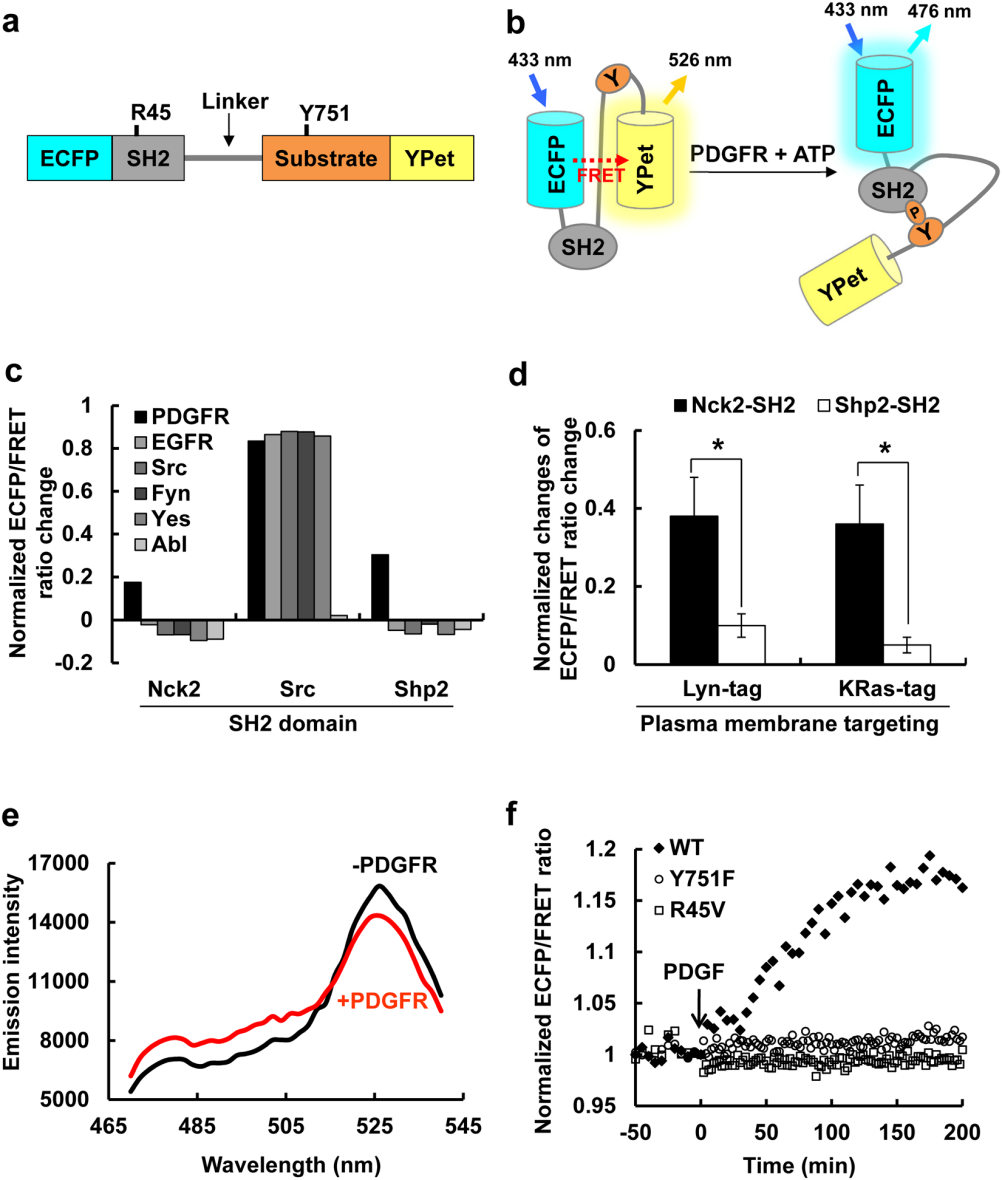
The design of the FRET-based PDGFR biosensor and its characterization. (**a**) The design of PDGFR biosensor composed of ECFP, a SH2 domain, a flexible linker, a substrate peptide containing PDGFR auto-phosphorylation site Tyr751, and YPet. (**b**) The PDGFR biosensor is designed to show strong FRET between ECFP and YPet, and this FRET will be reduced when active PDGFR phosphorylates the substrate at residue Y751 which will bind to the intramolecular SH2 domain, resulting in a conformational change and decrease in FRET. (**c**) The normalized ECFP/FRET ratio changes of PDGFR biosensors containing the SH2 domain from Nck2, Src or Shp2, in response to the treatment by different kinases: PDGFR, EGFR, Src, Fyn, Yes, or Abl as indicated. (**d**) The normalized ECFP/FRET ratio changes (mean ± s.e.m.) of membrane-bound PDGFR biosensors with Nck2-SH2 (black bars) or Shp2-SH2 (white bars) in live cells. The biosensors were targeted to the membrane microdomains by adding Lyn- or KRas tag for the continuous monitoring of PDGFR activation at membrane microdomains in live cells. (n = 10) * represents a significant difference between groups. (**e**) *In vitro* emission spectra of the purified PDGFR biosensor with Nck2-SH2 before (black line) and after (red line) the incubation with PDGFR. (**f**) The *In vitro* time courses of the PDGFR-mediated ECFP/FRET emission ratio of the purified Nck2-SH2 PDGFR biosensor (black diamond), and its mutants of Y751F in the substrate (white diamond) and of R45V in the SH2 domain (white circle). The basal ECFP/FRET emission ratios were normalized as 1 to conveniently visualize the general fold-change of FRET change after the addition of PDGFR.

The FRET-based PDGFR biosensor was designed to contain a SH2 domain, a flexible linker, and a specific substrate peptide sequence encompassing the auto-phosphorylation site Tyr751, which are concatenated between FRET donor ECFP and acceptor YPet (Fig. 1a). This PDGFR biosensor is expected to have a high level of FRET signals at rest, characterized by a strong emission signal from YPet at 527 nm when ECFP was excited at 433 nm (Fig. 1b left). As the activated PDGFR phosphorylates the substrate residue Tyr751, which then subsequently binds to the intramolecular SH2 domain, the conformation of the biosensors is expected to markedly change, resulting in the decrease of FRET signals and the increase of the ECFP emission at 476 nm (Fig. 1b right). Therefore, the PDGFR activation status can be visualized and quantified by calculating the emission ratio of ECFP/FRET, which is independent of the different expression levels of the PDGFR biosensors.

It has been reported that, upon PDGFR activation, the phosphorylated PDGFR can recruit multiple different SH2 domain-containing signaling molecules^1^. We hence created different versions of PDGFR biosensors containing different SH2 domains derived from Src, Nck2, or Shp2 to find the best SH2 domain for the PDGFR biosensor. When activated PDGFRs were applied to purified biosensors with different SH2 domains *in vitro*, the biosensor with the Src SH2 domain showed the largest change in the ECFP/FRET emission ratio (Fig. 1c, black bars). However, this version of PDGFR biosensor containing the Src SH2 domain also showed similarly strong FRET changes upon the addition of other kinases, e.g. EGFR, Src, Fyn, and Yes (Fig. 1c), suggesting that this PDGFR biosensor may not be specific for the selective detection of PDGFR activation. In contrast, PDGFR biosensors containing the SH2 domain from either Nck2 or Shp2 showed significant changes in ECFP/FRET ratio selectively upon PDGFR addition, but not other kinases examined (Fig. 1c).

To further examine these two versions of PDGFR FRET biosensors in live mammalian cells, we used two different lipid modification signals to guide the localization of the PDGFR biosensor at the membrane microdomains (Lyn-tag for lipid rafts or KRas-tag for non-raft microdomains)^8, 11^, and measured the PDGF-induced FRET responses of the biosensors containing SH2 domains from Nck2 or Shp2 in live MEF cells. As shown in Fig. 1d, the PDGFR biosensor with Nck2-SH2 domain shows much stronger FRET responses than the one with Shp2-SH2 domain in cells, with both membrane-targeting signals. Therefore, the PDGFR biosensor containing Nck2-SH2, which showed a high level of selectivity and sensitivity both *in vitro* and in live cells, was chosen as the FRET-based PDGFR biosensor.

We then further characterized the Nck2-SH2 PDGFR biosensor. A strong FRET signal (maximal at 526 nm) of the purified PDGFR biosensor was observed before the treatment of active PDGFR (Fig. 1e, black line). Upon the incubation of active PDGFR, the FRET signal decreased and the ECFP emission (maximal at 476 nm) increased (Fig. 1e, red line), confirming our designed FRET response of the PDGFR biosensor upon activation. The emission ratio of ECFP (at 476 nm) and FRET (at 526 nm) was calculated at each time point, and the basal level before the addition of PDGFR was normalized as 1 to display the general fold-change of FRET response more conveniently. This time course of *in vitro* kinase assay was shown in Fig. 1f. Because *in vitro* kinase assay is a kind of chemical reaction of PDGFR biosensor phosphorylation (substrate) by PDGFR kinase (enzyme) in the test tube, the response time of 2 hrs, which can largely depend on the molar ratio of substrate and enzyme as well as the reaction conditions, does not represent the real reaction time in the physiological condition. When key mutations either at the substrate (Y751F) or the SH2 domain (R45V) were introduced in the PDGFR biosensor, the addition of PDGFR did not cause any FRET change of the mutant biosensors (Fig. 1f), confirming that the *in vitro* FRET changes are derived from the intramolecular interaction between the phosphorylated Tyr in the substrate and the SH2 domain in the biosensor as we designed.

**Figure 2:**
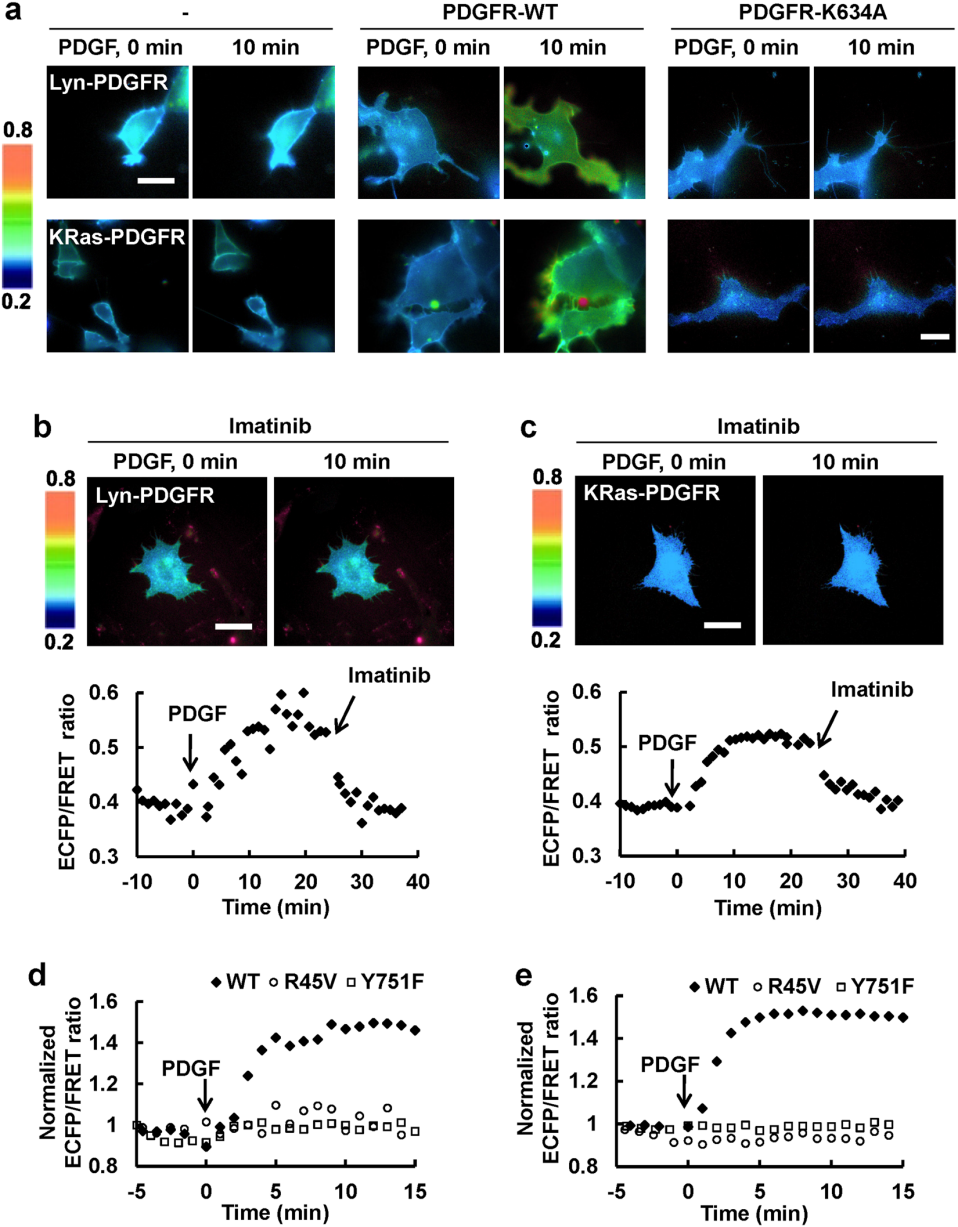
The specificity of the PDGFR biosensor in mammalian cells. (**a**) The representative ECFP/FRET emission ratios of the Lyn- and KRas-tagged Nck2-SH2 PDGFR biosensors before and after PDGF stimulation in PDGFR−/− MEFs with or without the reconstitution of wild type PDGFR or PDGFR-K634A mutant. The color bar on the left represents the values of ECFP/FRET emission ratio of PDGFR biosensor. Bar = 20 μm (**b** and **c**) The representative ECFP/FRET images of (**b**) Lyn- and (**c**) KRas-tagged PDGFR biosensors before and after PDGF stimulation in MEFs in the presence of the PDGFR-specific inhibitor Imatinib. Bar = 20 μm. The time courses of the ECFP/FRET ratios of Lyn- and KRas-PDGFR biosensors in the lower panels were results from MEFs subjected to PDGF treatment followed by the subsequent addition of Imatinib. (**d** and **e**) The time course of the normalized ECFP/FRET emission ratios of (**d**) Lyn- and (**e**) KRas-PDGFR biosensors. The PDGF-induced FRET responses of wild-type PDGFR biosensor (black diamond) as well as its mutants, R45V (in the SH2 domain, white circle) and Y751F (in the substrate, white square) were presented.

The selectivity of the PDGFR biosensor was further tested in mammalian cells. When the PDGFR biosensor with membrane targeting signal (either Lyn- or KRas-tag) was introduced in PDGFR−/− MEFs, no FRET response was detected upon the addition of PDGF (Fig. 2a). The introduction of wild-type PDGFR, but not the kinase-mutant (PDGFR K634A) in these PDGFR deficient cells allowed the PDGF-induced FRET response of both Lyn- and KRas-PDGFR biosensors (Fig. 2a). The FRET responses of Lyn- and KRas-PDGFR biosensors were also inhibited by the treatment of a PDGFR inhibitor, Imatinib (Fig. 2b and c). These results suggest that the FRET response of PDGFR biosensor can specifically report the PDGFR activation in live cells. In addition, the PDGF-induced FRET responses of both Lyn- and KRas-tagged PDGFR biosensors in cells were completely abolished with the key mutations either at the substrate (Y751F) or the SH2 domain (R45V) (Fig. 2d and e), confirming our designed FRET response of PDGFR biosensors from the intramolecular interactions between the substrate and SH2 domain in cells. Theses cellular FRET response started right after the addition of PDGF and the response started to be saturated after around 10 min (Fig. 2b–e), which is the same time range of PDGFR phosphorylation previously detected by western blotting assay^14^. Therefore, the newly developed PDGFR biosensor is sensitive enough to report the physiological PDGFR activation in live cells.

### PDGFR Is Activated Equally in and outside Lipid Rafts

The physicochemical properties of lipid rafts are different from other membrane regions. It has been suggested that different signaling molecules may be localized at different membrane mircodomains and the distribution of signaling molecules at membrane microdomains can be changed in response to physiological signals. It has been previously demonstrated that Lyn-tag, which contains N-terminal myristoylation (Gly) and palmitoylation (Cys) motifs, and KRas-tag, which includes the C-terminal polybasic residues (Lys) or farnesylation site (Cys), can target the protein cargoes selectively into or outside of lipid raft microdomains, respectively^6, 8, 11–13, 15^. We also confirmed the separate localization of Lyn- and KRas-tagged PDGFR biosensors at different membrane microdomains. As shown in the Supplementary Fig. S1a, the co-occurrence between the Lyn-PDGFR biosensor and cholera toxin subunit B (CT-B), which is known to selectively bind to the ganglioside GM1 located in lipid rafts^16, 17^, was greater than that between the KRas-PDGFR biosensor and CT-B and it was also clear that the KRas-PDGFR biosensor did not exclusively localize to the plasma membrane. As the size of lipid rafts is beyond the resolution of conventional microscope, it is technically difficult to clearly distinguish the differential localization of these biosensors. Thus, we next tried to separate Lyn- and KRas-PDGFR biosensors by sucrose density gradient membrane fractionation. The results show that Lyn-PDGFR biosensor, but not KRas-PDGFR biosensor, is in the upper fraction where the lipid raft marker CT-B is located (Supplementary Fig. S1b).

**Figure 3:**
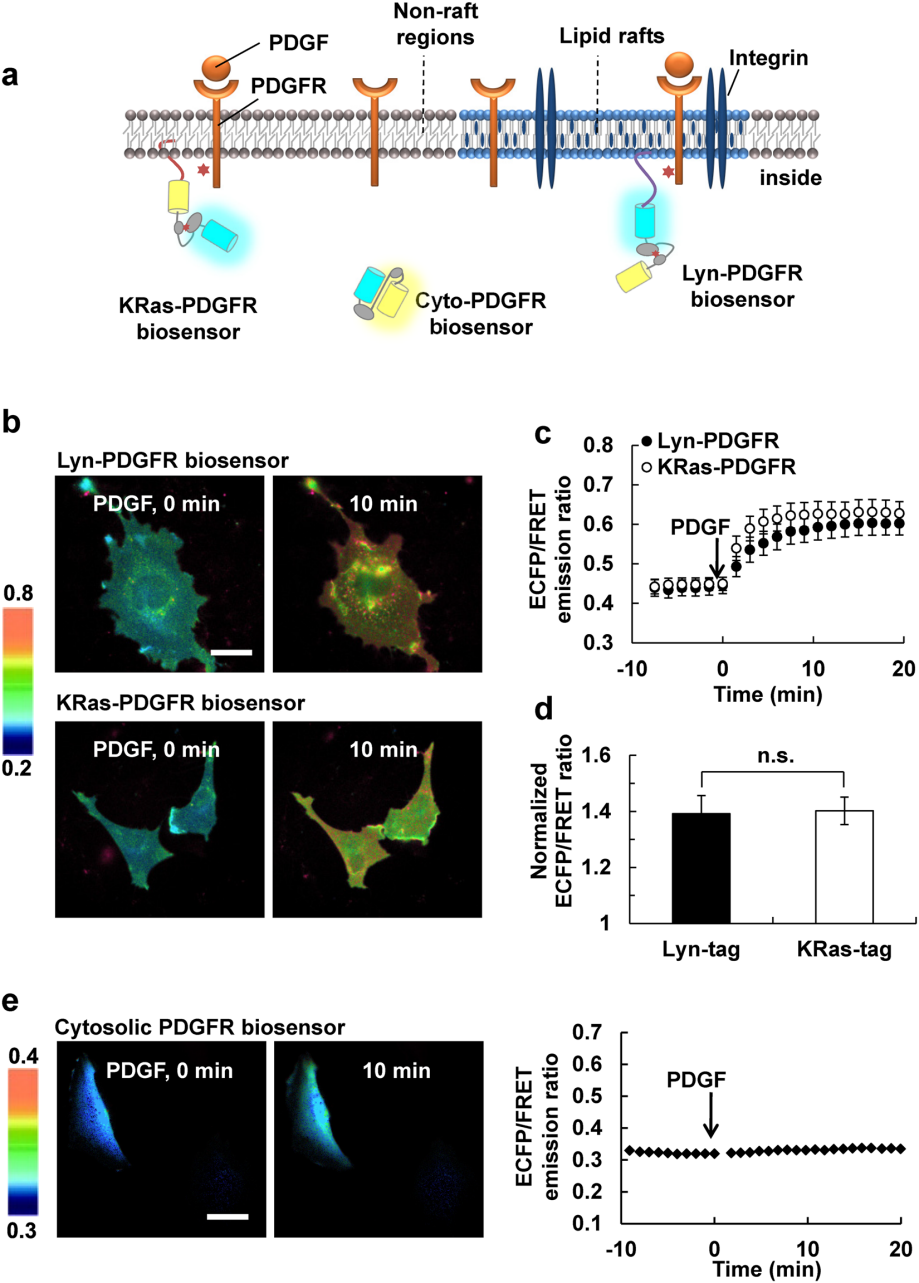
The PDGFR activation occurs both in and outside of lipid rafts. (**a**) The cartoon depicts the different subcellular localizations of cytosolic, Lyn- and KRas-tagged PDGFR biosensors. Lyn-tag contains the acylation signals for the incorporation of the biosensor into lipid rafts microdomains, while the prenylation signal of KRas-tag leads the biosensor to the general membrane regions outside of rafts. (**b**) The representative ECFP/FRET ratio images of Lyn- and KRas-PDGFR biosensors before and after 10 min of PDGF treatment in MEFs. The color bar on the left represents the values of ECFP/FRET emission ratio of PDGFR biosensor. Bar = 20 μm. (**c**) The time course of the ECFP/FRET ratio (mean ± s.e.m.) of Lyn- (black circles) and KRas-PDGFR biosensors (white circles) upon PDGF stimulation. (**d**) The normalized ECFP/FRET ratio at the time point of maximal FRET response from Lyn- (black) or KRas-tagged PDGFR biosensors (white) (n = 10). n.s. indicates no significant difference between groups. (**e**) The representative ECFP/FRET emission ratio images and time courses of the cytosolic PDGFR biosensor in response to PDGF treatment. Bar = 20 μm.

Therefore, we further utilized the PDGFR biosensors fused to the Lyn- and KRas-tags to investigate and compare the PDGFR activations at different membrane microdomains (Fig. 3a). When the FRET responses of Lyn- and KRas-PDGFR biosensors upon PDGF stimulation were compared, surprisingly, no significant difference was observed (Fig. 3b–d). In fact, in response to PDGF stimulation, MEFs expressing Lyn- and KRas-PDGFR biosensors showed similar changes in FRET responses. Cytosolic PDGFR biosensor with no targeting motif did not show a significant PDGF-induced change in FRET signals (Fig. 3e). These results suggest that the PDGF-induced PDGFR activation occurs quite strictly at the proximity of the membrane regions, both in and outside of the lipid rafts microdomains at similar levels.

### Antagonistic Effect of Integrin Signals on the PDGFR Activity in Lipid Rafts

**Figure 4:**
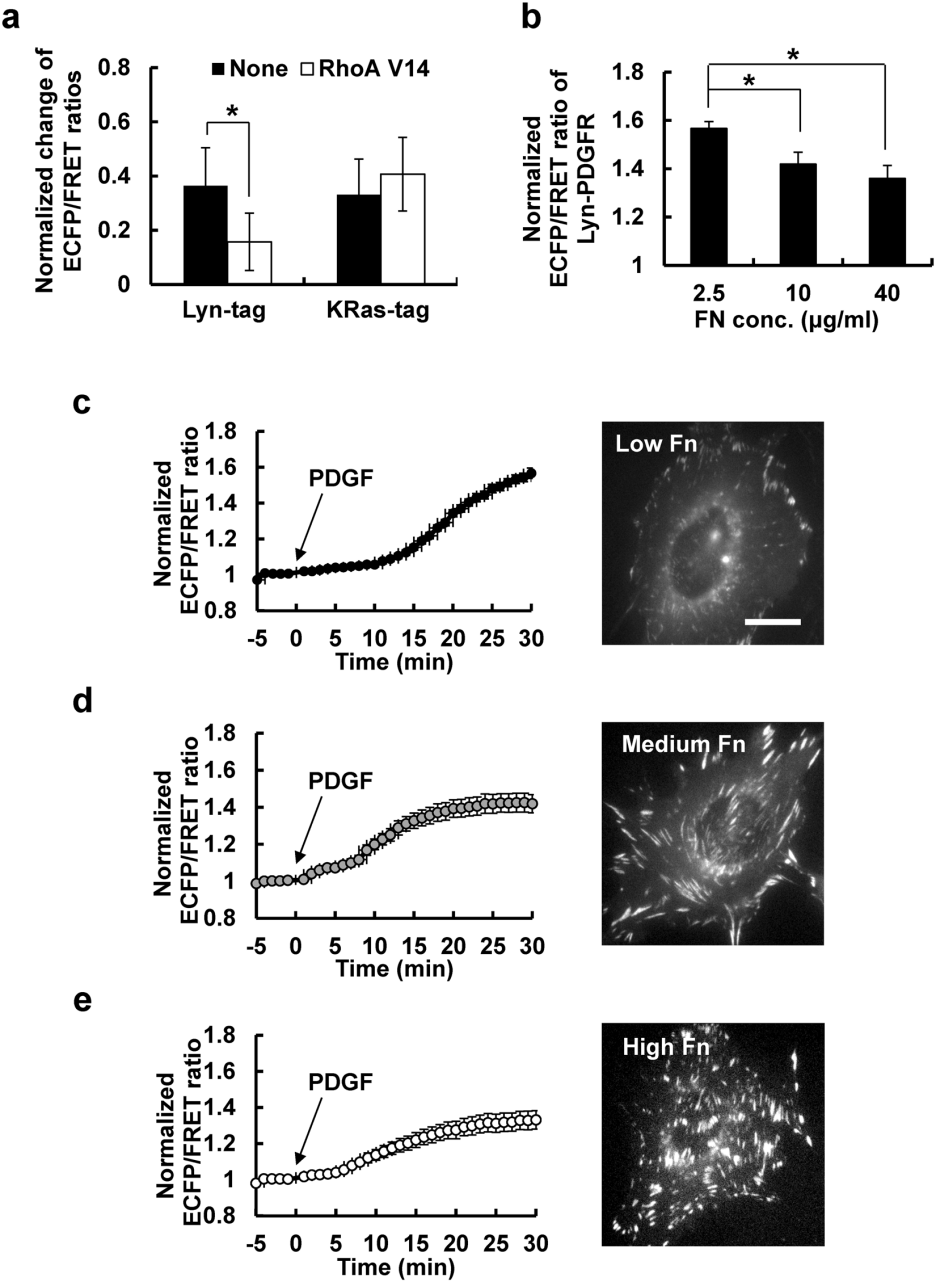
The PDGFR activation in lipid rafts, but not at non-raft membrane regions, is inhibited by integrin/RhoA-mediated cellular tension. (**a**) The normalized ECFP/FRET ratio changes of Lyn- and KRas-PDGFR biosensors with or without RhoA V14 expression in MEFs upon PDGF stimulation. (n = 6) (**b**) The comparison of maximal level of normalized ECFP/FRET ratios (mean ± s.e.m.). (n = 10) (**c**–**e**) The time courses of the normalized ECFP/FRET emission ratios of Lyn-PDGFR biosensor in response to PDGF stimulation. MEFs expressing the Lyn-PDGFR biosensor were plated on the dishes coated with different concentrations of fibronectin (Fn): 2.5 μg/ml (**c**), 10 μg/ml (**d**) or 40 μg/ml (**e**). The representative images of mCherry-tagged paxillin in MEFs cultured on different Fn concentrations are shown on the right. * represents significant difference between groups. (n = 10) Bar = 10 μm.

It has been suggested that matrix mechanics and cell tension, mainly mediated by integrin signaling, play important roles in cancer development and metastasis^18, 19^. Thus, we further tested the effect of integrin-mediated cellular tension on the PDGFR activation. We first checked whether the PDGFR activation in different membrane microdomains can be regulated by cellular tensional states induced by a constitutively active mutant of RhoA, which is a key effector related to integrin-regulated intracellular tension. Introducing this mutant RhoA V14 significantly decreased the PDGF-induced changes in the ECFP/FRET emission ratio of Lyn-tagged PDGFR biosensors, but not KRas-tagged PDGFR biosensor (Fig. 4a). These results indicate the PDGFR activation at lipid rafts is more sensitive to the RhoA-regulated cellular tension.

To further assess the effect of integrin-induced cellular tension on the PDGFR activation at lipid rafts, we prepared the dishes coated with different concentrations of fibronectin (Fn), which will cause different levels of integrin activation^20^, then compared the FRET changes of Lyn-PDGFR biosensors upon PDGF stimulation on these dishes. Our results showed that the overall FRET change is significantly higher on the low Fn group (2.5 μg/ml) compared to the ones on medium Fn (10 μg/ml) or high Fn (40 μg/ml) groups (Fig. 4b,c–e left panels). In fact, different Fn concentrations caused different levels of integrin-related downstream events, e.g. degree of focal adhesion assembly (Fig. 4c–e right panels) and FAK activation (Supplementary Fig. S2). The basal FRET levels of Lyn-tagged PDGFR biosensor before the PDGF stimulation were not significantly different in three groups (Supplementary Fig. S3). As low Fn concentrations should trigger weaker integrin related signaling comparing to that of high Fn concentration^20^, these results suggest an antagonistic effect of integrin-induced cellular tensions on the PDGFR activation at the membrane microdomains lipid rafts.

## Discussion

PDGFR is a transmembrane receptor, which recognizes and engages its ligand PDGF via the extracellular domain and transfers the signal into the cells by a clustering-mediated auto-phosphorylation at its intracellular tail which further recruits SH2-containing signaling molecules. It remains controversial on whether PDGFR is located at lipid rafts microdomains, which are suggested to be involved in the sorting of specific proteins during signal transduction. For example, previous studies^21, 22^ reported that PDGFR is located in a specific type of lipid rafts called caveolae, and in response to PDGF treatment, the phosphorylated tyrosine-containing proteins are recruited to transduce signaling in these structures. In contrast, it has also been suggested that the scaffolding domain of caveolin, a key protein in caveolae, has an inhibitory role in PDGFR signaling^23, 24^. These results suggest a limitation of the previous methodologies to study accurate signaling pathways at the dynamic lipid raft structures. In fact, the separation of lipid raft membrane fractions is largely affected by the types of detergent and the treatment condition, and nonspecific effects of methyl-β-cyclodextrin have been critical issues in this field^3–5^.

In the current study, we developed a novel PDGFR biosensor based on the fluorescence resonance energy transfer (FRET) to investigate the dynamic PDGFR activity in different membrane microdomains. The PDGFR biosensor includes a substrate containing Tyr751 of PDGFR, a major autophosphorylation site^25, 26^ (Fig. 1a and b). As a phosphotyrosine-binding domain of the biosensor, different SH2 domains from Nck2, Src, and Shp2 were tested using various kinases, e.g. PDGFR, EGFR, Src, Fyn, Yes, and Abl (Fig. 1c). While the Src-SH2 containing biosensor presented generally larger FRET changes in response to all the kinases tested, the biosensors including Nck2-SH2 or Shp2-SH2 showed the better selectivity toward PDGFR (Fig. 1c). When these two versions of PDGFR biosensors were further compared in mammalian cells, Nck2-SH2 containing biosensor showed a better response to PDGF (Fig. 1d), thus selected as a novel PDGFR FRET biosensor. The FRET response of this newly developed PDGFR biosensor was specific to the PDGFR activation *in vitro* and in mammalian cells as confirmed by spectrum assay, and by using PDGFR mutants and a PDGFR inhibitor Imatinib (Figs 1f and 2). Therefore, we have successfully developed a FRET-based PDGFR biosensor, which will allow the real-time visualization of PDGFR activation with high spatiotemporal resolutions in live cells.

It has been previous trials to measure the concentration of PDGF utilizing aptamer-functionalized gold nanoparticle^27^, and to detect the interaction of PDGFR and its downstream signaling molecules using bioluminescence resonance energy transfer (BRET) technology^28^. However, the aptamer-based PDGF sensor is more appropriate for the *in vitro* detection of PDGF concentration in solutions rather than for the study of PDGF-induced intracellular signaling pathways inside a live cell^27^. The BRET-based assays between the PDGFR-Rluc (Renilla luciferase, serving as a donor) and the acceptor GFP-attached downstream molecules such as Grb2 and PLCγ1, are useful to confirm the signaling events in cells^28^, but requires complicated analysis given different expression levels and distributions of these interacting molecules. Furthermore, these assays cannot provide the quantitative information of different subcellular activity of PDGFR activation with high spatiotemporal resolutions. Thus, the FRET-based PDGFR biosensor in this study can provide a novel tool to investigate the real-time subcellular PDGFR activation in live cells with high sensitivity and specificity.

To investigate the subcellular activity of PDGFR at membrane microdomains, different lipid modification sequences, e.g. acylation or prenylation, were fused to the PDGFR biosensor. It has been confirmed that prenylation signals from KRas can target a protein to the general membrane regions outside lipid rafts, while acylation signals derived from Lyn kinase are sufficient to target a protein into lipid rafts^29^. FRET biosensors with different lipid modifications have been used as powerful tools to differentiate dynamic protein activities at different membrane microdomains in live cells^6^. In particular, the PDGF-induced Src activation was stronger outside lipid rafts^9, 11^ while FAK and Akt activation upon PDGF stimulation was stronger in lipid rafts^8, 12, 13^. Surprisingly, our studies utilizing Lyn- and KRas-PDGFR biosensors discovered no significant difference in the PDGFR activation in and outside lipid rafts (Fig. 3a), suggesting that the PDGFR-mediated signaling events may occur both in and outside lipid rafts as well as at the plasma membrane and intracellularly since the KRas-PDGFR biosensor was not exclusively found at the plasma membrane. These results thus verify the previous results reporting different activities of Src, FAK, and Akt at membrane microdomains in response to PDGF^8, 11–13^ are mainly due to the differential distribution of these downstream signaling molecules, but not that of the PDGFRs. The non-raft targeting signal derived from KRas4B, which was widely used in previous studies^6–8, 11–13, 29–32^, has been suggested to be sensitive to the phosphorylation mediated by a strong PKC agonist^33^, thus careful attention is required when used in PKC-related signaling study.

Another important finding in the current study is that the integrin-mediated downstream events, represented by strong focal adhesions with high FAK and RhoA activities, have an inhibitory role in the PDGF-induced activation of PDGFR, specifically in lipid rafts, but not at general membrane regions outside lipid rafts (Fig. 4). These data suggest an antagonistic effect of integrin-related signaling on the PDGFR activation specifically in lipid rafts. It has been previously shown that the integrin-mediated RhoA activity, at focal adhesions, inhibits the PDGFR-induced Rac1 activity and ROS production^34^. As close links between focal adhesions and lipid rafts have been suggested^35^, this previous report also supports the current work suggesting an antagonistic relationship between integrin and PDGFR signals at the specialized rafts microdomains.

Our results are particularly interesting because previous studies reported synergic functions of integrin and PDGFR. For example, it has been shown that, in the absence of PDGF, integrin clustering itself can induce the phosphorylation of PDGFR^36^. Another study confirmed that integrin aggregation and occupancy can synergistically promote the phosphorylation of PDGFR upon PDGF stimulation^37^. In particular, the extracellular domain of integrin subtype β3 was shown to directly bind to PDGFR^38^, and the association of PDGF-induced activated PDGFR and integrin αvβ3 potentiated the PDGFR-mediated biological functions, such as cell adhesion and migration^39, 40^. However, these studies investigated the overall effects on the PDGF-induced downstream events, either in the presence or absence of integrin signals, but did not assess the effect of different strengths of the integrin-mediated signaling events. Hence, the current study is complementary to these previous studies and revealed that strong integrin signaling and cellular tension can play inhibitory roles on the PDGFR activation, specifically at lipid rafts.

This antagonistic function of integrin signaling on the PDGFR activation at lipid rafts may be due to the caveolin proteins existing at lipid rafts, whose scaffolding domain can directly bind to the proximal kinase domain of PDGFR and inhibit the activation of PDGFR^23, 24^. Because integrin clustering and focal adhesion signaling are suggested to be focused in highly ordered lipid rafts structure^41^, and stronger integrin-related signals may result in the larger size/structure of lipid rafts as well as the increased level of caveolin proteins incorporated in lipid rafts. Thus, the lipid rafts-specific antagonistic effect of integrin signals may be because of the increased inhibitory functions of caveolin proteins on the PDGFR activation (Supplementary Fig. S4). Alternatively, caveolin-induced internalization of growth factor receptors^42^ can contribute to the inhibition of PDGFR activation at lipid rafts.

Taken together, the novel FRET-based PDGFR biosensors targeted at membrane microdomains have great advantages to continuously report the real-time PDGFR activity at membrane microdomains in live cells with high spatiotemporal resolutions. Our approach with these FRET biosensors revealed tightly regulated PDGFR activities at subcellular levels depending on the extracellular environments and intracellular signals. As PDGFR plays important roles in many essential cellular processes such as cell proliferation, migration and survival, these findings will advance our in-depth understanding on the molecular mechanism underlying these pathophysiological processes.

## Methods

### DNA Construction and Plasmids

The cytosolic PDGFR biosensor (Cyto-PDGFR) was constructed by fusing the SH2 domain, a flexible linker (GSTSGSGKPGSGEGS), and a specific substrate including the PDGFR auto-phosphorylation site Y751 (DESVD**Y**VPMLDM) between the N-terminus ECFP and the C-terminus YPet. The SH2 domain from Nck2, c-Src, or Shp2 was amplified by polymerase chain reaction (PCR) and inserted in the construct to create PDGFR biosensor with different SH2 domains. After initial tests, we mainly used the SH2 domain derived from Nck2 for the rest of the study.

The PDGFR (Y751F) and (R45V) mutant biosensors were generated by site-specific mutations on the substrate tyrosine site (Y751F) and the critical arginine site (R175V) in the SH2 domain, respectively, using the QuickChange method (Stratagene). Lyn-PDGFR biosensor was constructed by adding a raft-targeting motif (M**GC**IKSKRKDNLNDDE) derived from Lyn kinase to the N-terminus of the Cyto-PDGFR biosensor. KRas-FAK biosensor was constructed by adding a non-raft-targeting motif (KKKKKKSKTK**C**VIM) derived from KRas to the C-terminus of the Cyto-PDGFR biosensor. The DNA encoding the FAK biosensors were subcloned with the BamHI/EcoRI sites in pRSetB for the protein purification from *Escherichia coli*, and in pcDNA3 plasmid for the expression in mammalian cells.

RhoA V14 was described previously^43^. PDGFR wild type or mutant at K634A are gifts from Prof. Hamid Band at University of Nebraska Medical Center.

### Cell Culture and Reagents

Mouse embryonic fibroblasts (MEFs) and human embryonic kidney (HEK) cells were maintained in Dulbecco’s modified Eagle medium (DMEM) supplemented with 10% fetal bovine serum (FBS), 2 mM L-glutamine, 1 unit/ml penicillin, 100 μg/ml streptomycin, and 1 mM sodium pyruvate. Cell culture reagents were purchased from GIBCO BRL. Cells were cultured in a humidified 95% air, 5% CO_2_ incubator at 37 °C. Lipofectamine 2000 (Invitrogen) was used for the transfection of plasmids.

Platelet-derived growth factor (PDGF), fibronectin (Fn), and methyl-β-cyclodextrin (MβCD) and Imatinib were purchased from Sigma.

### Protein Expression and in vitro Kinase Assay

The PDGFR biosensors were expressed in *Escherichia coli* (BL21 strain) as fusion proteins with an N-terminal 6x *His* tag and purified by nickel chelation chromatography^7^. Fluorescence emission spectra of the purified biosensors were measured with an excitation wavelength of 430 nm by a fluorescence plate reader (TECAN, Sapphire II). The emission ratios of donor/acceptor (478 nm/526 nm) were measured before and after the addition of 1 mM ATP and 1 μg/ml of PDGF receptor (Millipore), EGF receptor (Sigma), Src (Upstate Biotechnology), Fyn (Upstate Biotechnology), Yes (Upstate Biotechnology), or Abl (Calbiochem) in kinase buffer (50 mM Tris·HCl, 100 mM NaCl, 10 mM MgCl_2_, 2 mM DTT, pH 8)^7^.

### Image Acquisition

Cells were cultured in cover-glass-bottom dishes (Cell E&G) and maintained in CO_2_-independent medium containing 0.5% FBS (Gibco BRL) at 37 °C during imaging. Images were collected by a Zeiss Axiovert microscope and MetaFluor 6.2 software (Universal Imaging) with a 420DF20 excitation filter, a 450DRLP dichroic mirror, and two emission filters controlled by a filter changer (475DF40 for ECFP and 535DF25 for YPet). The excitation filter for ECFP at 420 ± 20 nm was selected to shift toward lower wavelength away from the peak excitation spectra of ECFP to reduce the cross-excitation of FRET and the effect of bleed-through on the FRET channel. Because the membrane-targeted FRET biosensors showed similar FRET responses in different regions across the whole cell area under epi-fluorescence microscope setting, the whole cell body of the target cell expressing the FRET biosensor was selected as a region of interest (ROI) to collect signals and conduct quantification. The fluorescence intensity in the background region was selected and quantified to subtract the signals from the ROI of ECFP and FRET channels. The pixel-by-pixel ratio images of ECFP/FRET were calculated based on the background-subtracted fluorescence intensity images of ECFP and FRET as follows.

$$\frac{I(ECF{P}_{ROI})-I(ECF{P}_{background})}{I(FRE{T}_{ROI})-I(FRE{T}_{background})}$$

where *I* represents the intensity of each region from each channel as indicated.

These ratio images were displayed in the intensity modified display (IMD) mode in which the color and brightness of each pixel is determined by the ECFP/FRET ratio and ECFP intensity, respectively.

### Statistical analysis

Each representative image and graph were achieved after at least three independent experiments. *P* values were calculated using student’s t test and an asterisk indicates *p* < 0.05.

## Electronic supplementary material

Supplementary Information with Supplementary Figures
