# Supplementary material for: FRET-based Visualization of PDGF Receptor Activation at Membrane Microdomains
Source: Sci Rep. 2017 May 9;7:1593. doi: 10.1038/s41598-017-01789-y (PMC5431615; doi:10.1038/s41598-017-01789-y)

FRET-based Visualization of PDGF Receptor Activation at Membrane Microdomains

Jihye Seong[1-4*], Min Huang[5], Kyoung Mi Sim[2], Hyunbin Kim[2,4], Yingxiao Wang[1,5,6*]

[1]Neuroscience Program, University of Illinois, Urbana-Champaign, Urbana, IL, 61801, USA, [2]Center for Diagnosis Treatment Care of Dementia, Korea Institute of Science and Technology (KIST), Seoul, 02792, Korea, [3]Biological Chemistry Program, Korea University of Science and Technology (UST), Daejeon, 34113, Korea, [4]Department of Converging Science and Technology, Kyung Hee University, Seoul 02447, Korea, [5]Department of Bioengineering, University of Illinois, Urbana-Champaign, Urbana, IL 61801, [6]Department of Bioengineering, University of California, San Diego, CA 92093, USA

***To whom correspondence should be addressed.**

Jihye Seong, Ph.D.
Center for Diagnosis Treatment Care of Dementia, Korea Institute of Science and Technology (KIST), Seoul, 02792, South Korea
Tel: +82-2-958-5904
Fax: +82-2-958-5189
E-mail: jseong@kist.re.kr

Yingxiao Wang, Ph.D.
Department of Bioengineering, University of California at San Diego, La Jolla, CA 92093-0435
Tel: +1-858-822-4502
Fax: +1-858-822-1160
E-mail: yiw015@eng.ucsd.edu

Keywords: PDGF receptor, FRET biosensor, Lipid rafts, Integrin, Intracellular Tension

Running Title: FRET-based visualization of PDGFR activation

Supplementary Methods

Lipid Rafts Imaging with Cholera Toxin-B

Cells expressing the Lyn- or KRas-PDGFR biosensor were washed with pre-chilled media and incubated with 1 μg/ml cholera toxin subunit B (recombinant) labeled with the Alexa Fluor 594 (Molecular Probes) for 10 min at 4℃. After the incubation, cells were gently washed several times with pre-chilled PBS, then further incubated with anti-CT-B antibody (1:200, Molecular Probes, Cat#: V-34405) for 15 min at 4℃. After the incubation, cells were gently washed several times with pre-chilled PBS and fixed with 4% formaldehyde for 15 min at 4℃. The cells were washed several times with PBS, and then imaged by fluorescence microscope.

Sucrose Density Gradient Membrane Fractionation

MEFs expressing the Lyn- or KRas–PDGFR biosensor were rinsed twice with ice-cold PBS and lysed with TNE buffer (10 mM Tris, pH 7.4, 150 mM NaCl, and 5 mM EDTA) containing 2 mM PMSF, 2 mM NaF, 2 mM $NaVO_4$, 10 nM Caluculin A, and protease inhibitor cocktail tablet. Then, the cells were subjected to mechanical disruption with 15 strokes of a homogenizer. Homogenates were centrifuged at 2,300×g for 5 min at 4℃, and the supernatant was centrifuged at 18,000×g for 50 min at 4℃. The resulting membrane pellets were resuspended in 2.5 ml of 1% Triton X-100 containing TNF buffer supplemented with 2 mM PMSF, 2 mM NaF, 2 mM $NaVO_4$, 10 nM Caluculin A, and protease inhibitor cocktail tablet. These crude plasma membranes were incubated in ice with periodic mixing for 1 h, then diluted 1:1 with 80% sucrose and layered on 10 mL of 35% sucrose, followed by the addition of 2.5 mL of 5% sucrose solution and 11.25 mL of

TNE buffer. Ultracentrifugation was performed at 141,000×g for 18 h in a Beckman SW28 rotor. All experimental steps were performed at 4℃. After ultracentrifugation, the top 8.75 mL of the sample was discarded. Different membrane fractions were then collected, starting from the top of the gradient. The fractions were dot-blotted on nitrocellulose membranes and probed with Alexa Fluor 594-conjugated cholera toxin subunit B to identify the raft-containing fractions[1].

Supplementary Figure Legends

Supplementary Figure S1. (**a**) The representative images of cholera toxin subunit B (CT-B) conjugated with Alexa Fluor 594 and subsequently crosslinked with CT-B antibody (Molecular Probes, left panels), Lyn- or KRas-PDGFR biosensors (middle) and the merged images of CT-B and biosensors (right). Bar=20μm. (**b**) The MEF cells overexpressing Lyn- or KRas-PDGFR biosensors were subjected to sucrose density gradient membrane fractionation, and the localization of the biosensors were detected by western blotting with an anti-GFP antibody. The fractions of lipid rafts were confirmed by dot blotting with CT-B.

Supplementary Figure S2. The representative ECFP/YPet emission ratio images (**a**) and the average values (mean ± s.e.m.). Bar=10μm (**b**) of FAK biosensors[2] at basal levels in HT1080 cells cultured on the different fibronectin concentrations. (n=22-24) * represents significant difference between groups as indicated.

Supplementary Figure S3. The basal ECFP/FRET ratios of the Lyn-PDGFR biosensors on different Fn concentrations as indicated. (n=10). n.s. indicates no significant difference between groups.

Supplementary Figure S4. A proposed model of antagonistic effect of integrin/RhoA-induced cellular tensions and caveolins on the PDGFR activation in lipid rafts.

References

1 Gao, X. *et al.* PI3K/Akt signaling requires spatial compartmentalization in plasma membrane microdomains. *Proc Natl Acad Sci U S A* **108**, 14509-14514, doi:10.1073/pnas.1019386108 (2011).

2 Seong, J. *et al.* Detection of focal adhesion kinase activation at membrane microdomains by fluorescence resonance energy transfer. *Nat Commun* **2**, 406, doi:10.1038/ncomms1414 (2011).

Supplementary Figure S1

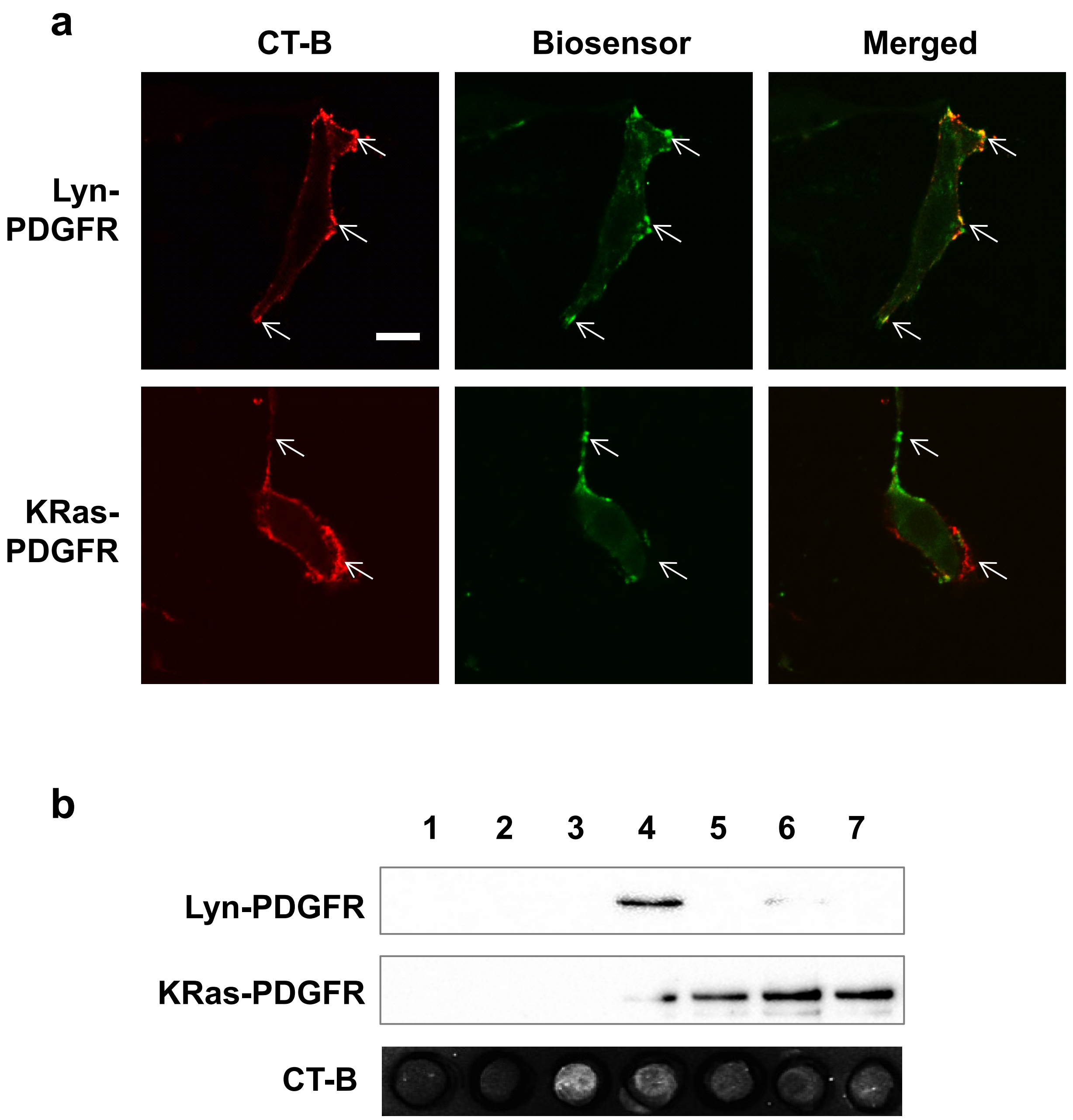

Supplementary Figure S2

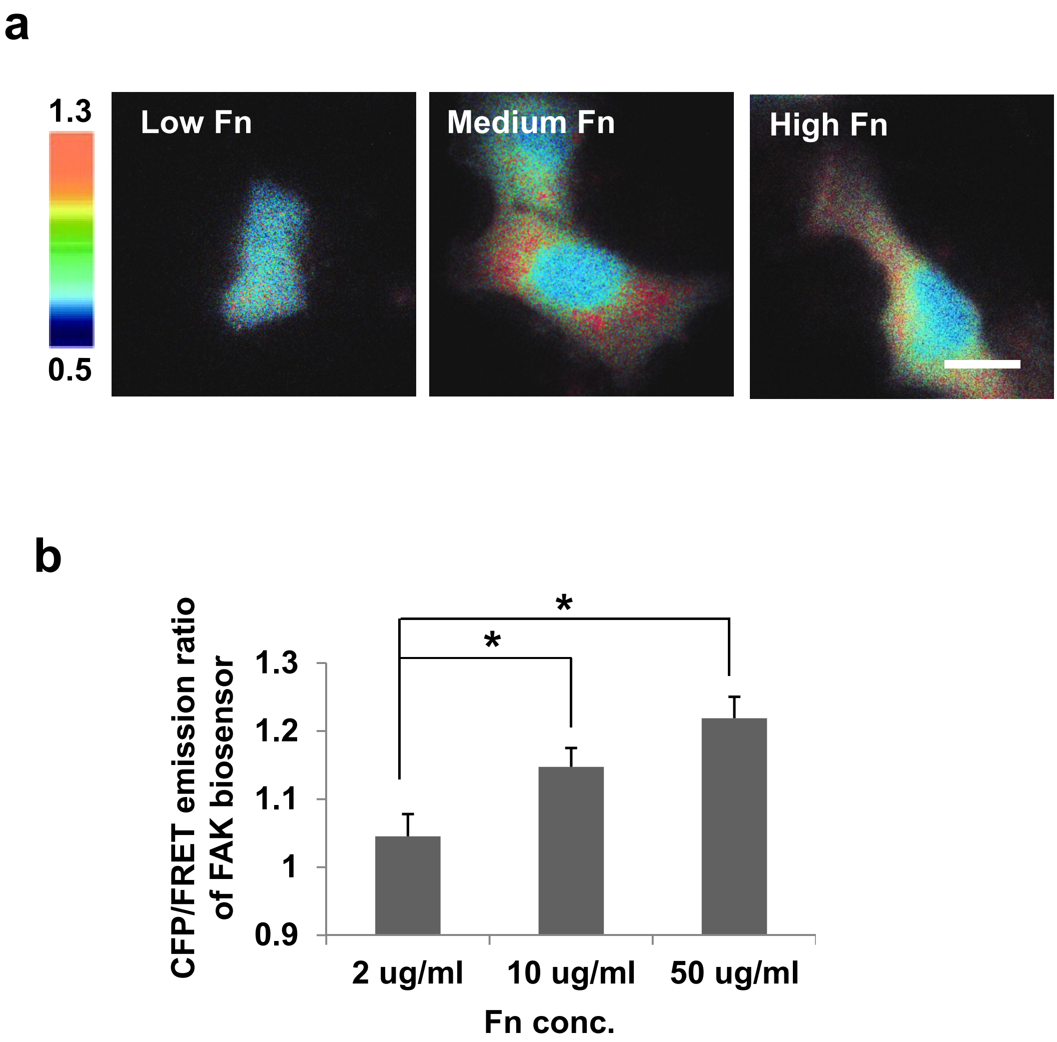

Supplementary Figure S3

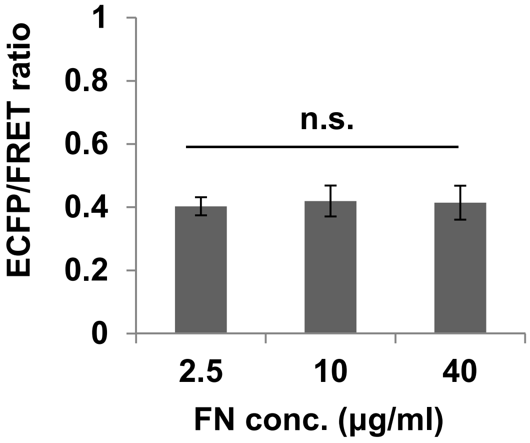

Supplementary Figure S4

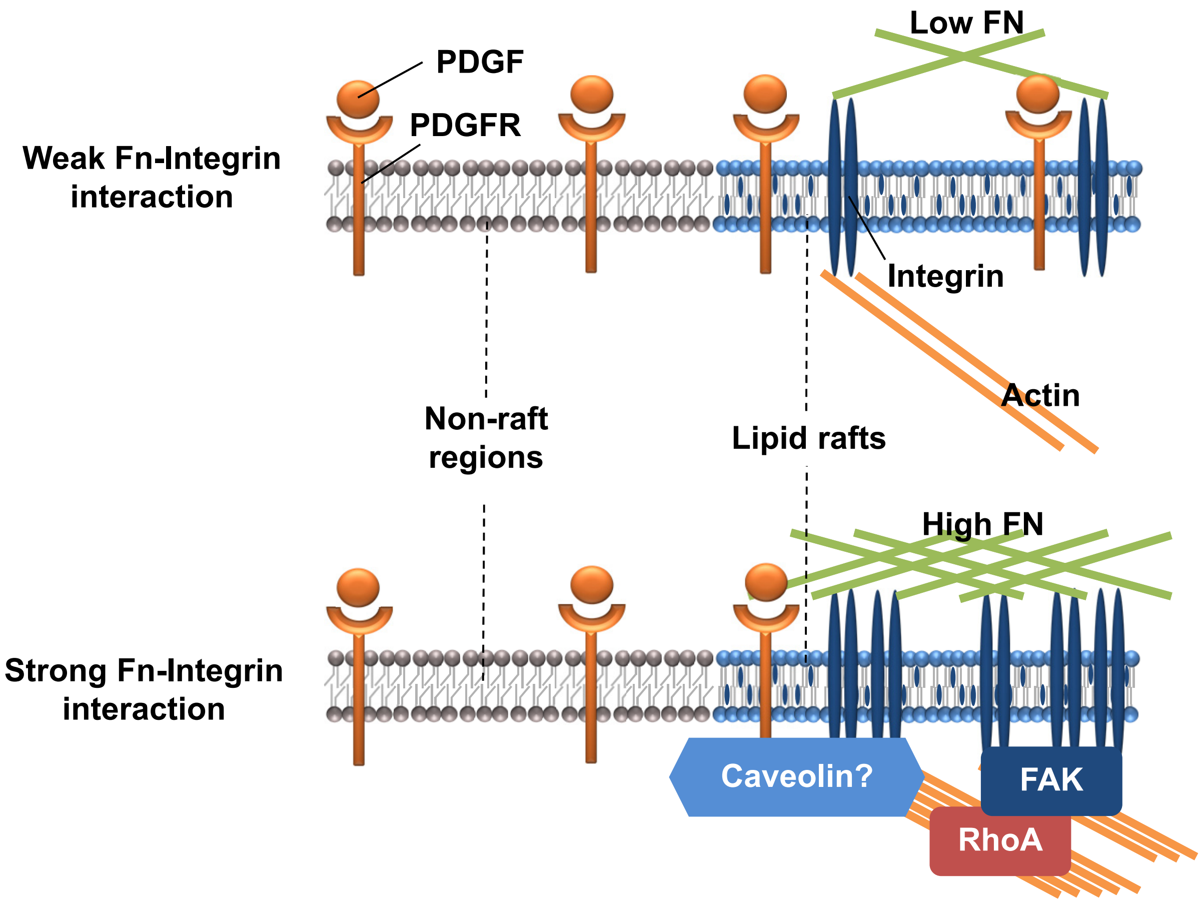

Supplement: Supplementary file 1 — Supplementary Information with Supplementary Figures [file 41598_2017_1789_MOESM1_ESM.pdf]
